# An Hybrid Approach for Urban Traffic Prediction and Control in Smart Cities

**DOI:** 10.3390/s20247209

**Published:** 2020-12-16

**Authors:** Janetta Culita, Simona Iuliana Caramihai, Ioan Dumitrache, Mihnea Alexandru Moisescu, Ioan Stefan Sacala

**Affiliations:** Faculty of Automatic Control and Computers, Politehnica University of Bucharest, 060042 Bucharest, Romania; janetta.culita@upb.ro (J.C.); simona.caramihai@upb.ro (S.I.C.); ioan.dumitrache@upb.ro (I.D.); ioan.sacala@upb.ro (I.S.S.)

**Keywords:** urban traffic prediction, smart city, neuro-inspired control architecture, complex systems, parametric model

## Abstract

Smart cities are complex, socio-technological systems built as a strongly connected System of Systems, whose functioning is driven by human–machine interactions and whose ultimate goals are the well-being of their inhabitants. Consequently, controlling a smart city is an objective that may be achieved by using a specific framework that integrates algorithmic control, intelligent control, cognitive control and especially human reasoning and communication. Among the many functions of a smart city, intelligent transportation is one of the most important, with specific restrictions and a high level of dynamics. This paper focuses on the application of a neuro-inspired control framework for urban traffic as a component of a complex system. It is a proof of concept for a systemic integrative approach to the global problem of smart city management and integrates a previously designed urban traffic control architecture (for the city of Bucharest) with the actual purpose of ensuring its proactivity by means of traffic flow prediction. Analyses of requirements and methods for prediction are performed in order to determine the best way for fulfilling the perception function of the architecture with respect to the traffic control problem definition. A parametric method and an AI-based method are discussed in order to predict the traffic flow, both in the short and long term, based on real data. A brief comparative analysis of the prediction performances is also presented.

## 1. Introduction

Smart cities represent one of the major challenges addressed to systems engineering from the perspective of the complexity and diversity of control objectives for the constituent (sub)systems.

They may be seen as a system of systems because many of their functions (heating, illumination, transportation, communication, health) are performed by dedicated technological systems that are strongly interconnected (water networks, gas networks, electricity grids [1], power systems) and which may and should be reconfigured in order to ensure the proper global functioning. The impact of malfunctioning of a local subsystem component may affect the functioning of the whole through a decision chain that involves not only technological systems but especially human reasoning.

This is because smart cities are naturally human-oriented [2]; they are built for human well-being, offering support for a wide range of applications or services [3] that aim to enhance people’s quality of life [4]. Thus, in [5], the smart cities focus on the people (by integrating the key dimensions of culture, metabolism and governance) prior to adopting modern information communication and technology (ICT) (artificial intelligence (AI), big data and the Internet of things (IoT)). Moreover, every technological system/machine interacts with people and must adjust their functioning to human needs. Human to machine, machine-to-machine and human-to-human interaction and communication are key elements in the structure and functioning of a city, thus making it a complex socio-technological system [6,7].

The recent focus in smart city research addresses relevant technology integration, such as the Internet of things (IoT)–cloud application [3,8,9,10] that integrates AI and big data paradigm [4,11]. In this context, a holistic approach of the smart city from an integrative systemic perspective of complex systems is relevant [12]. Controlling complex systems is a goal that may be approached either by taking into account the specificity of each case and address it in a particular way, by networking, interfacing and integration of subsystems or by a systemic view, which implies the development of a generic modeling framework.

This paper’s main purpose is to apply a generic neuro-inspired framework designed for the control of complex systems to the problems of a smart city, focusing, as a proof of concept, on urban traffic, as one of the main integrative subsystems of the city [13].

It addresses and integrates a control architecture that was previously designed by the authors’ research team to be used in the city of Bucharest and, in the process, allows improving its functioning by the perception function. Integrating already existent control subsystems, especially by perception and sense, is one of the main future research developments that are intended for the neuro-inspired control architecture.

More precisely, the paper addresses the way in which traffic data can be used and interpreted so as to allow the reconfiguration of the system in relation to the number of vehicles (traffic flow).

The urban traffic analysis can be referred to in various ways, usually relying on numerical information as car flow (the number of cars passing on a road segment over a fixed period), traffic density (in a certain area), car speed [11,14]. These type of data, collected, in general, by means of specialized devices (sensors, video cameras), can be interpreted and numerical modeled by using the concept of time series [15,16]. In dynamic and complex systems, like urban traffic, accurate traffic prediction is not easy to achieve, especially when the measured data are affected by stochastic disturbances (noises) that usually come from the environment. In urban traffic, the environment noise can be associated with several factors [17]: the moment of the day, unfavorable weather conditions, undesirable events, social events, road repairing works, changing the traffic route of drivers, a day before holidays, the weekends before winter holidays, starting the classes (school).

This paper deals with predicting car flow variations in the short and long term. Predicted traffic values provide a decisional base for reconfiguring the traffic control architecture. More precisely, based on the predicted car flow, the local junction controllers set the traffic light duration of the corresponding traffic light in order to avoid junction congestion.

Two important research directions are commonly reported in traffic prediction literature [17,18,19]: one based on parametric models (or statistic-based) and one based on nonparametric (nonconventional or AI-based) models [17,20]. Lately, due to the increased quantity of data and the computation capability [14], most contributions have been focusing on the AI-based approach, more specifically, the machine learning/deep learning (support vector regression (SVR), k-nearest neighbor, Bayesian Network, random forest, convolutional neural networks CNN, recurrent neural network RNN, graph CNN, long-short-term-memory LSTM), reinforcement learning and transfer-learning methods [11,14,17,21,22,23,24].

Although the superiority of DL-based methods for traffic forecasting has been substantially documented [14,18,20,25], parametric methods can still provide good results for specific cases. In this context, this paper aims to investigate and compare the prediction performances of both types of methods and to discuss their applicability in a real urban traffic scenario based on data collected from the city of Bucharest. For the second approach, a DL based model, namely long short-term memory neural network (LSTM-NN), was adopted.

The rest of the paper is structured as follows: the Methodology section is focused on presenting the problem definition, the essential features of the neuro-inspired approach and of the urban traffic control architecture and a short description of the two prediction methods. Section 3 and Section 4 present a case study based on real data and comparatively discuss and analyze the prediction performances achieved by the two methods. Some concluding remarks are provided in Section 5.

## 2. Methodology

### 2.1. Some Considerations on Problem Definition

The control approach is based on a neuro-inspired architecture [26], which is built on the similitude between certain classes of complex systems and the human body.

The human body has, indeed, different levels of resolution, from cell to organ and then to the global organism; each of them is a part of a highly networked system and contributes to the emergent functioning of the organism. The malfunction of one component may affect several subsystems and usually results in the reconfiguration of the overall system in order to fulfill the global functioning requirements.

Its control system, the brain, has developed so that it can manage this complexity by different granularity levels and by appropriate management of the sensorial information that ensures an equilibrium between qualitative and quantitative representations and the focus on the most relevant inputs. Decisions are based both on locally obtained data (through own sensorial systems) and on communication with similar systems; selection/optimization criteria are both objective (quantitative) and subjective (emotions).

A smart city also has different levels of granularity—from atomic agents as people and local control systems to networked systems as the power system [1,27], the traffic system [28], the health system [27,29]. There are many decisions involving data obtained through local sensors and information acquired by communication with other subsystems [13]. Being in nature a social system, many of the behaviors and decisions are based on both objective and subjective criteria.

Actually, smart/intelligent mobility is one of the most important functions of a smart city [28], with a strong impact on the well-being of the city inhabitants, and thus, urban traffic control is one of the core functionality in a control architecture of a smart-city. In addition to the urban traffic system being interconnected with practically all the important subsystems of a smart-city, it combines strong restrictions (as the traffic infrastructure capacity—streets, junctions) with high dynamics both concerning the vehicles flow and the human driver decisions and behaviors. In this respect, there are large levels of uncertainty in model building as well as considerable delays in the propagation of a control decision, which requires a proper prediction function to support the control system.

The approach to such systems must be inherently hybrid in nature (qualitative/quantitative) and address all levels of granularity simultaneously.

In addition, from an engineering point of view, it is important to identify models of “good practice” for the control of systems of similar complexity, to be used as a basic approach for designing an appropriate control framework: architecture, operating principles, control levels, etc. (top-down approach). This framework must then be applied and validated in a bottom-up manner that allows the synthesis of the final control system.

The aspects to be solved when defining a neuro-inspired framework for complex systems are, mainly [26,30,31,32,33]:

- the granularity levels of control (from measurement towards searching, grouping and focusing data in order to obtain qualitative models);
- the reasoning approaches (from implicit and quantitative—embedded algorithms—to explicit and qualitative) for problem-solving;
- the learning methods/validation of solutions;
- behavior-generation methods.

From the granularity point of view and following the human brain similitude, we are defining the following levels:

- reflex actions—local control, based on (embedded) algorithms and based on sensorial data or their interpretation through dedicated interfaces;
- learned behavior—(offline) designed and tested scenarios, based on adaptive and multimodel control structures; they are triggered by specific conditions and usually replace reflex actions in order to acquire optimal results;
- strategic problem-solving—qualitative approach for new problems, based on trial and error system reconfiguration to be solved by learned behaviors.

This kind of architecture has taken into consideration complex systems that may be addressed as interconnected autonomous subsystems having largely heterogeneous physical characteristics—as cyber-physical systems. When the same ontology is used on every cyber-physical system, their integration may be achieved by using a common knowledge management system and a common set of goals—which was illustrated on intelligent cyber-enterprises [26,33].

In this paper, the actual complex system to be dealt with is a smart city, decomposable by its functional subsystems: power, electricity, urban traffic, smart transportation, a.s.o.

We have chosen for testing the validity of our approach an urban traffic control architecture that was previously designed and partially implemented by our research team for the city of Bucharest, and that was tested on real data and which was updated for the purpose [34,35,36].

### 2.2. The Urban Traffic Control Architecture—Brief Description

The city of Bucharest is one of the most crowded cities with respect to the traffic infrastructure and the increasing number of vehicles. The city developed during the last 30 years in a mostly chaotic way, driven by real estate opportunities. Large residential units were developed in sub-urban regions, without an appropriate development of traffic infrastructure and public transportation. A large majority of residents use their own car for daily transport, both to go to work and for shopping and to take their children to school—the best of which are in the city center. The downtown traffic infrastructure remained essentially unchanged since 1990 when the number of personal automobiles has increased ten times.

This is the reason for steps have been taken to implement an intelligent traffic control system whose main objective has been to avoid congestion [34].

The control architecture was designed having in mind open-system integration capabilities, on three levels, of which the lower level was implemented and tested. The three levels were: junction level control; network-level supervision, strategic traffic control.

From the neuro-inspired architecture point of view, junction level control is the level of the reflex actions. A control system was associated with every junction, based on an agent-oriented hybrid Petri net model approach to calculating, based on measured data and structural modeling, the appropriate duration of traffic lights in order to ensure the fact that the length of waiting/input vehicles queue does not exceed the capacity of the street. This is a process of continuous adaption of the traffic light duration to the traffic flow through the predicted values.

**Figure 1:**
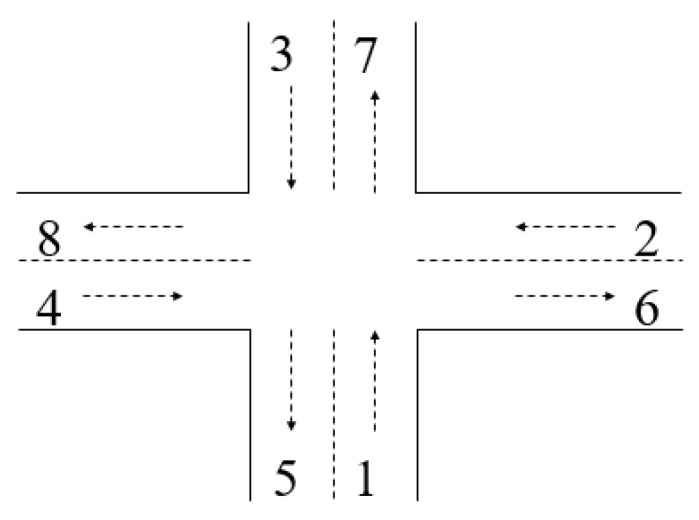
X-junction with a 3-phase light scenario.

One of the key aspects in such an approach is the structure of the traffic light scenario (order of traffic lights and overall duration of a cycle; adaptation to the traffic context was made by changing the duration of every light, respecting the order and the duration of the cycle—which were denominated as a light scenario). Figure 1 shows an example of an X-junction with a 3-phase light scenario (specification: cycle duration 120 time-units), where phase 1 is associated with connections: 1–6, 1–7, 1–8, 3–8, 3–5, 3–6 (green), phase corresponds to the connections 2:4–5, 4–6, 4–7 (green) and phase to the connections 3:2–7, 2–8, 2–5 (green).

**Figure 2:**
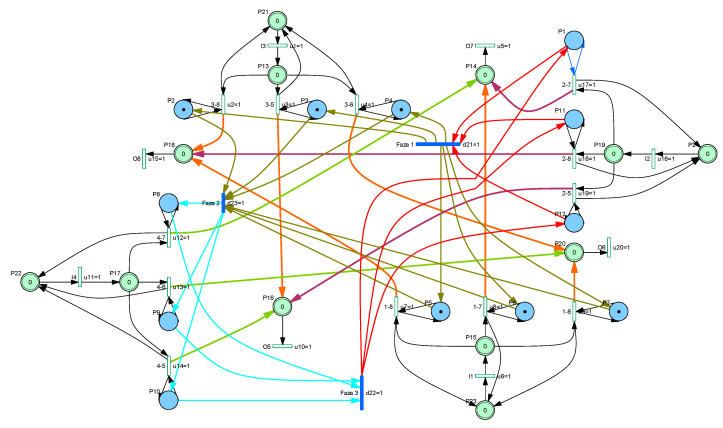
Hybrid Petri net model of the junction in Figure 1.

Figure 2 is presenting the hybrid Petri net that is modeling this junction, where, based on the initial marking in continuous places—received from sensors placed on the input lanes—are computed, for every light cycles, the durations that should be associated with every timed transition in order to ensure the emptying of input queues.

The problem which was identified was that the constant parameters allowed adaptation only for a given traffic structure (density of vehicles); hence the reason to change them when appropriate. As changing the duration of a traffic light cycle in a junction affects adjoining junctions as well, eventually creating congestion, the problem was to be solved by the network supervising level.

Here, the supervision level is monitoring a group of adjoining junctions (a subnet of the traffic network) and identifies the situations in which a certain junction light scenario becomes obsolete, changing it with another, form a library of offline prepared and tested ones, and eventually adapting also the light scenarios of the adjoining junction. This is the level of learned behavior.

The third level will be concerned with strategical decisions, as establishing the subnet structure to be managed by a given supervisor (which may change dynamically with respect to the density of the overall traffic and to the state of the system), identifying traffic concentrators and events that may necessitate the reconfiguration of the traffic system a.s.o.

The key aspect for the problem to be solved at the supervision level is to correctly identify the traffic structure that requires a change of the light scenario, as the functional requirement for the control architecture to correctly use learned behavior, which involves permanent monitoring. The rest of the paper is dealing with the development of procedures allowing this functionality.

The control architecture, as it was implemented ([35,36]), is reactive at the level of junction control and may adapt for a given range of car-flow density. When this range exceeds a given limit, however, the traffic light scenario must be changed, not only for a junction but for a subnet of adjoining junctions. The appropriate values of car-flow density, as well as traffic light scenarios, are offline computed.

However, it is important for the overall functioning of the control architecture to precisely determine the moment in which to change the scenario, thus ensuring the pro-active quality of the traffic control system. This functionality is essential for the learned-behavior level of the neural approach and represents a secondary objective of this paper.

The functioning cycle of the control architecture is:

- for a given light scenario: real-time adaptation of traffic light duration and traffic flow monitoring
  ○ until the occurrence of a threshold event (car flow density reaches a predefined level, requiring the light scenario exchange)
    ▪ exchange traffic light scenario according to offline calculus; adjust parameters of the junction models
    ▪ implement a new scenario

As the adjustment of scenarios exceeds a light cycle, which could lead to congestion, it is important to predict the moment where the actual scenario should be changed at least three light cycles before the occurrence of the actual event.

This is the reason for the prediction of certain traffic thresholds is taken into account as an objective of this paper.

As previously mentioned, two modeling approaches are studied in view of short and long-term urban traffic prediction: parametric and nonparametric. The structure of the parametric models is determined by theoretical considerations [14] based on quadratic criterion minimization, while the structure (and parameters) of the AI-based models are determined from data [14]. A summary description of these models applied to time series modeling and prediction is introduced in the sequel.

### 2.3. The Parametric Model

As well known, a time series is a sequence of data, chronological ordered, acquired from a process or natural phenomenon during its evolution. In general, any time series integrates two types of behaviors: one deterministic and one non-deterministic (stochastic). In practice, it is considered that the prediction model of the time series results from the sum of the two behavior models. The deterministic component is based on a trend and a seasonal variation. The trend shows a general orientation of the data (an average), while the seasonal variation emphasizes the repetitive nature of data if any. The non-deterministic component models the stochastic environment noises.

In the classic approach, all three components are described by parametric models and can be estimated by means of the system identification/signal processing techniques [16], as shortly explained in the sequel.

Denote a times series of length $N\in {\mathbb{N}}^{*}$ by ${\left\{y[n]\right\}}_{n=\bar{1,N}}$ (or simply y) where n are the sample instants. The trend component is modeled by a generic polynomial

(1) $$y_{T}[n]=\alpha_{0}+\alpha_{1}n+\cdots +\alpha_{r}n^{r},\;\forall \;n\in \bar{1,N}$$

where the coefficients ${\left\{\alpha_{i}\right\}}_{i\in \bar{0,r}}$ and the polynomial degree r are the unknown parameters of the trend model. Assuming the degree r is known, the optimal coefficients can be determined by applying the least-squares method (LSM) [15,16] that minimizes a quadratic criterion based on the prediction error between the observed data y and the trend model $y_{T}$. One must mention that the trend model must be parsimonious (with a small degree, e.g., 3), but sufficient to discriminate it from the other two components (seasonal and stochastic) with good accuracy. Otherwise, the trend could incorporate the seasonal variation and, moreover, the noises that affect the data.

The seasonal component modeling takes into account a time–frequency approach. It starts from the stationary data, obtained by subtracting the trend $y_{T}$ from the observed data y. Generally, the seasonal model $y_{S}$ is described by a number P (the period) of seasonal coefficients ${\left\{y_{S,p}\right\}}_{p\in \bar{1,P}}$ that are replicated by periodicity over the measurement horizon. However, a time series can present several seasonal variations with different periods. In order to determine these periods, the temporal Whittaker–Robinson method can be applied successively [16] in conjunction with the minimization of the square errors between $y_{S}$ and the current stationary series till no seasonal variation is detected anymore. Then, the seasonal variation can be expressed as a Fourier series with the previously detected periods. The optimal seasonal (Fourier) coefficients are estimated by means of a frequency method (based on Schuster periodogram and LSM) [16].

Finally, by subtracting the seasonal component from the stationary data, one obtains the stochastic component ${\left\{v[n]\right\}}_{n\in \bar{1,N}}$, which has a noisy character. The stochastic component can be modeled by an autoregressive (AR) and moving average (MA) (ARMA) model:

(2) $$\left\{ \begin{array}{l} A\left(q^{-1}\right)v[n]=C\left(q^{-1}\right)e[n]\\ E\left\{e[n]e[m]\right\}=\lambda^{2}\delta_{0}[n-m] \end{array}\right.,\;\forall \;n\in \bar{1,N}$$

In (2) $q^{-1}$ stands for the one-step delay operator ($\left(q^{-1}f\right)[n]=f[n-1]$,∀ n∈ℤ), e is Gaussian white noise with unknown variance $\lambda^{2}$, while A and C are polynomials of degrees na and nc, respectively:

(3) $$\left\{ \begin{array}{l} A\left(q^{-1}\right)=1+a_{1}q^{-1}+\cdots +a_{na}q^{-na}\\ C\left(q^{-1}\right)=1+c_{1}q^{-1}+\cdots +c_{nc}q^{-nc} \end{array}\right.$$

If the structural indices of the model (2) are known, the coefficients of the ARMA model can be estimated by using the minimum prediction error method (MPEM) [15]. After estimating the polynomials in (3), the simulated stochastic component $y_{\mathrm{ARMA}}$ results from the system below [17]:

(4) $$\left\{ \begin{array}{l} y_{\mathrm{ARMA}}[n]=-{\hat{a}}_{1}y_{\mathrm{ARMA}}[n-1]-\cdots -{\hat{a}}_{na}y_{\mathrm{ARMA}}[n-na]+{\hat{c}}_{1}\hat{e}[n-1]+\cdots +{\hat{c}}_{nc}\hat{e}[n-nc]\\ \hat{e}[n]=v[n]+{\hat{\beta }}_{1}v[n-1]+\cdots +{\hat{\beta }}_{n\beta }v[n-n\beta ] \end{array}\right.,\;\forall \;n\in \bar{1,N}$$

where the symbol $\hat{\cdot }$ stands for the estimated parameters of the ARMA model (after using MPEM). Accordingly, $\hat{e}$ is the estimation of the white noise obtained by using an approximant AR model of order nβ, which has to be set much bigger than max{na,nc}(e.g., min{3max{na,nc},⌈N/3⌉}). A natural initialization of the recursive procedure (4) is: $y_{\mathrm{ARMA}}[n]=v[n]$, $\forall \;n\in \bar{1,\max\{na,nc\}}$. Finally, the estimation of the residual white noise (i.e., the global model error) is obtained by:

(5) $$\varepsilon [n]=v[n]-y_{\mathrm{ARMA}}[n],\;\forall \;n\in \bar{1,N}$$

As well known, the smaller the variance of the model error, the more accurate the stochastic model (and the entire parametric model).

In order to optimize the structure of the deterministic and stochastic models, for prediction purposes, usually one can apply optimization criteria related to the model error dispersion evaluated on the entire or a reduced observed dataset [37]. Finally, both models have to be estimated based on the entire available dataset. The optimal deterministic model is obtained by varying the polynomial degree r in a small range (e.g., 0÷5), and the optimal ARMA model can be built by varying the structural indices na and nc in a range of randomly (but uniformly) generated values. Obviously, the exhaustive search of the optimal indices should be avoided due to the time constraints.

After estimating the optimal component models, one can predict the time series. Denote by [N+1…N+K] the prediction horizon, where K is the number of predicted values. While the deterministic components are forecasted by simply extrapolating the two signals ($y_{T}$ and $y_{S}$) on the prediction horizon, the predicted values of the stochastic model ARMA ${\hat{y}}_{ARMA}$, are estimated by iterative application of the equations (4) [16,37,38] that take into account either the noise values v (on the measured horizon) or the previously estimated values of the stochastic model on the prediction horizon ${\hat{y}}_{ARMA}$:

(6) $$[\begin{array}{l} {\hat{y}}_{ARMA}[N+k]=-{\hat{a}}_{1}{\hat{y}}_{ARMA}[N+k-1]-\cdots -{\hat{a}}_{k-1}{\hat{y}}_{ARMA}[N+1]-{\hat{a}}_{k}v[N]-\cdots -{\hat{a}}_{na}v[k-na]+\\ \;+{\hat{c}}_{1}\hat{e}[N+k-1]+\cdots +{\hat{c}}_{nc}\hat{e}[N+k-nc];\\ \hat{e}[N+k]={\hat{y}}_{ARMA}[N+k]+{\hat{\beta }}_{1}{\hat{y}}_{ARMA}[N+k-1]+\cdots +{\hat{\beta }}_{k-1}{\hat{y}}_{ARMA}[N+1]\\ \;+{\hat{\beta }}_{k}v[N]+\cdots +{\hat{\beta }}_{n\beta }v[N+k-n\beta ]; \end{array}$$

where $\forall \;k\in \bar{1,K}$ and K<min(na,nβ).

Finally, the predicted values of the time series resulting from:

(7) $$\hat{y}[N+k]=y_{T}[N+k]+y_{S}[N+k]+{\hat{y}}_{ARMA}[N+k],\;\forall \;k\in \bar{1,K}$$

For the sake of simplicity, in the rest of the paper, the parametric model will be referred to as the ARMA model.

### 2.4. The Deep Learning Model

Deep learning (DL) techniques are a subclass of AI-based algorithms designed to observe similar behaviors and dependencies (patterns) in the processed data in order to build prediction (regression) or classification models without prior programming. These models are considered nonparametric. Basically, DL algorithms work as follows: having a data set containing training examples, as well as the corresponding outputs/labels of these examples, they learn the correspondence between the processed example and its output by creating a model and then apply what they have learned (i.e., the model) to the new data, whose output is not previously known. The approximation degree (fitness) between the yielded output and the real one determines the performance of the model. Obviously, the larger the training set, the better the accuracy.

Recurrent neural network (RNN) is a class of nonparametric model suitable for time series prediction since their recurrent hidden units are able to correlate the previous data to the current one based on the learned pattern. Still, they are unable to learn the long-term dependencies between the time steps of sequence data due to the vanishing gradient problem that significantly slows down the learning process. This shortcoming is eliminated by an advanced version of RNN, named the long short-term memory neural network (LSTM-NN) [14,39], where the recurrent units have been replaced with memory cells. Each cell is integrated into a memory block that has the role to selectively store the relevant information over a long period and to forget the irrelevant one, by means of three types of gates/filters (forget, input and output), the corresponding activation functions and some mathematical operators [20].

A typical architecture of LSTM-NN for time series prediction consists of four layers: the sequence input layer, the LSTM layer, the fully connected layer and the regression layer. For a simple time series, there is a single sequence input layer and a fully connected layer. The LSTM layer contains a variable number of memory blocks (equivalent to hidden units) and represents the core of the architecture.

When working with deep learning models for time series prediction, the measured datasets must be partitioned into two subsets: the training subset on which the learning process is carried on and the test subset that serves for evaluating the prediction quality. The LSTM network performance depends on the values of some hyper-parameters that must be primarily set: the number of hidden units and the training parameters (e.g., the maximum epoch number, the minimum batch size, the initial learning rate, etc.). Finding the optimal values of the hyper-parameters is based on training data and can be carried out by several technics: trial end error, random search, grid search or Bayesian optimization.

In order to evaluate the urban traffic prediction accuracy, several statistical metrics can be employed [14,18,39]: mean absolute error (MAE), mean squared error (MSE), root mean squared error (RMSE), mean average percentage error (MAPE). Almost all metrics take into account the prediction error that represents the difference between the real data (or test data in the AI-based approach) and the predicted data. In this paper, we employed the root-mean-square error (RMSE) and mean absolute error. (MAE) defined as follows:

(8) $$RMSE=\sqrt{\frac{1}{N}\sum_{n=1}^{N}{\left(e_{n}\right)}^{2}}$$

(9) $$MAE=\frac{1}{N}\sum_{n=1}^{N}\left|e_{n}\right|$$

where ${\left\{e_{n}\right\}}_{n=\bar{1,N}}$ is the prediction error of length N (evaluated on the prediction horizon). Obviously, the lower the values of these metrics, the higher the prediction accuracy.

## 3. Results

Our research is oriented towards the traffic issues of the city of Bucharest, one of the most crowded cities and whose infrastructure is barely adequate for the number of existing cars.

**Figure 3:**
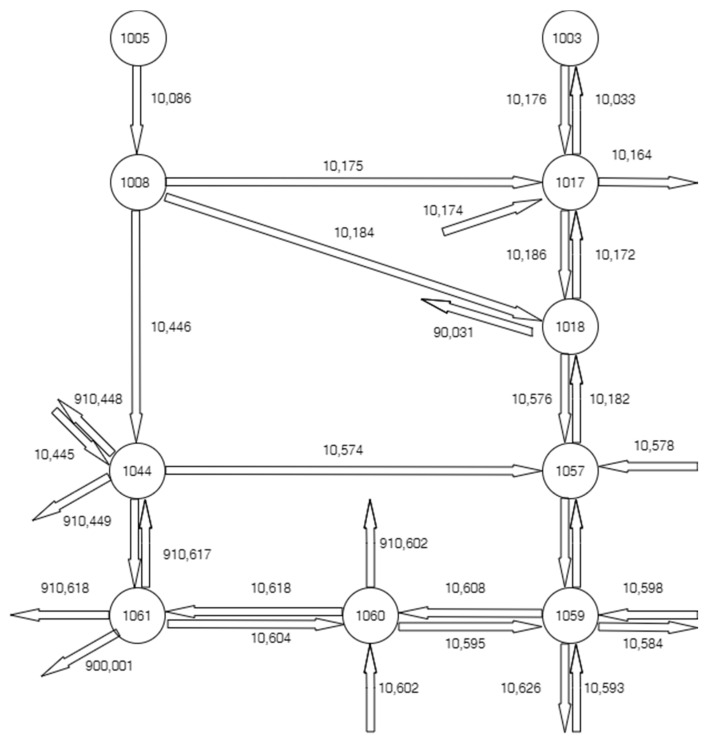
Study area.

The case study presented in this paper is based on real-world data gathered from a small part of a larger area of our city that contains several routes and cross streets, as in Figure 3. For better understanding and explanations, each entity is referred by an ID number. The main objective is to avoid traffic congestion by adjusting the phase durations of the traffic lights that control each crossroad according to the real traffic conditions. In order to prevent early traffic jams on a certain route (composed of consecutive segments), one desires to predict the traffic information on short (25 min) and eventually longer (50 min) terms, based on previously acquired data.

Bucharest has been facing critical traffic issues for a long time, mainly due to the undersized infrastructure with respect to the number of cars. The parallel routes presented in Figure 3 are important roads that bind the north region to the south and are crowded the most part of the (working) days. There are several other reasons for traffic jams, especially during rush hours (e.g., within 7–9 h), typically related to people’s habits or their car dependence. For example, in the northern zone of the city, there are placed more offices compared to the southern zone, but there are also more expensive houses. Therefore, many people live in the southern zone and work in the opposite zone, being forced to cross the city center every working day. Another cause for an overcrowded city center is that most people prefer to take their children to the city center schools by car daily (not only on rainy days), on their way to the office.

Based on the predicted traffic information, different traffic scenarios (functioning regimes) can be envisaged. For example, if the predicted values of the traffic data exceed a certain threshold, the traffic light functioning regime can switch on an appropriate one. Thus, the duration of the traffic light phase can be adaptively set according to the selected regime whenever it is necessary. Moreover, predicting traffic information can help the drivers in choosing an alternative, less crowded route for their destination. For example, if the route composed of 10,086, 10,446 and10,616 is overloaded and the parallel route composed of 10,576 and 10,596 is more available, the driver can be advised to opt for the latter one.

A functioning regime is mainly characterized by the traffic flow, but weather conditions, social events and traffic events can be very helpful to improve the traffic prediction accuracy as proved in [39].

**Figure 4:**
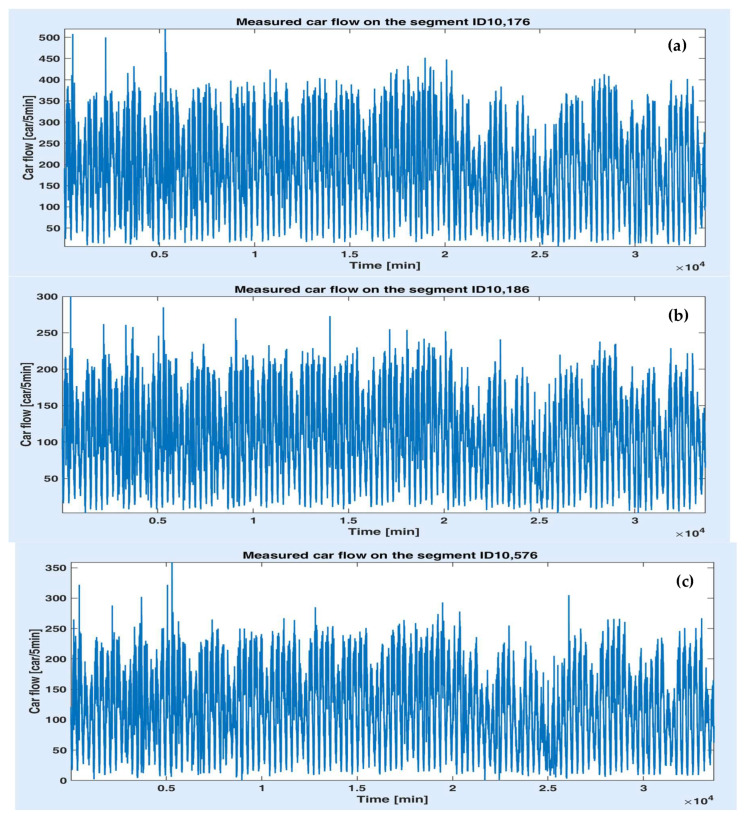
Car flow variation on the segments ID 10,176 (**a**), 10,186 (**b**) and 10,576 (**c**) on October–January.

In this paper, the available traffic information consists of the car flow (number of cars per 5 min) collected on the segments denoted by ID 10,176, 10,186 and 10,576, respectively. The datasets were acquired by means of a video camera on the period of 1 October–31 January with a 5 min time step. Figure 4a–c depicts the measured car flow on the segments ID 10,176, 10,186 and 10,576. Although the data on all segments seem to have a periodic variation overall, their evolution is quite irregular and strongly disturbed. Therefore, obtaining an accurate prediction is a challenging task. Some statistical features of the measured data sets are presented in Table 1. One notes that the variance (or the standard deviation) has high values.

**Figure 5:**
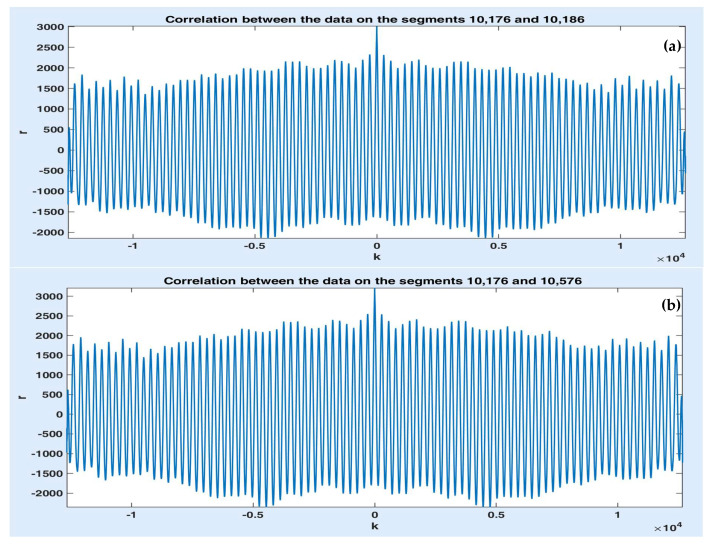
Correlation between data of segments 10,176 and 10,186 (**a**) and of segments 10,176 and 10,576 (**b**).

As noticed in Figure 4, the data on the three segments are correlated, which should lead to correlations between the predicted values, as well. In this case, there is no need to monitor and predict all the consecutive segments of the same road since the traffic scenarios can be set by the predicted value of the first segment only. This is an important aspect since one can simplify the communications system structure that contains traffic tracking devices, avoiding supplementary costs and efforts. Figure 5a,b illustrates the correlation sequence between the data on the first two segments and between data on the first and the third segment. One observes that these functions have high amplitudes and are very oscillating, which proves the strong correlation.

**Figure 6:**
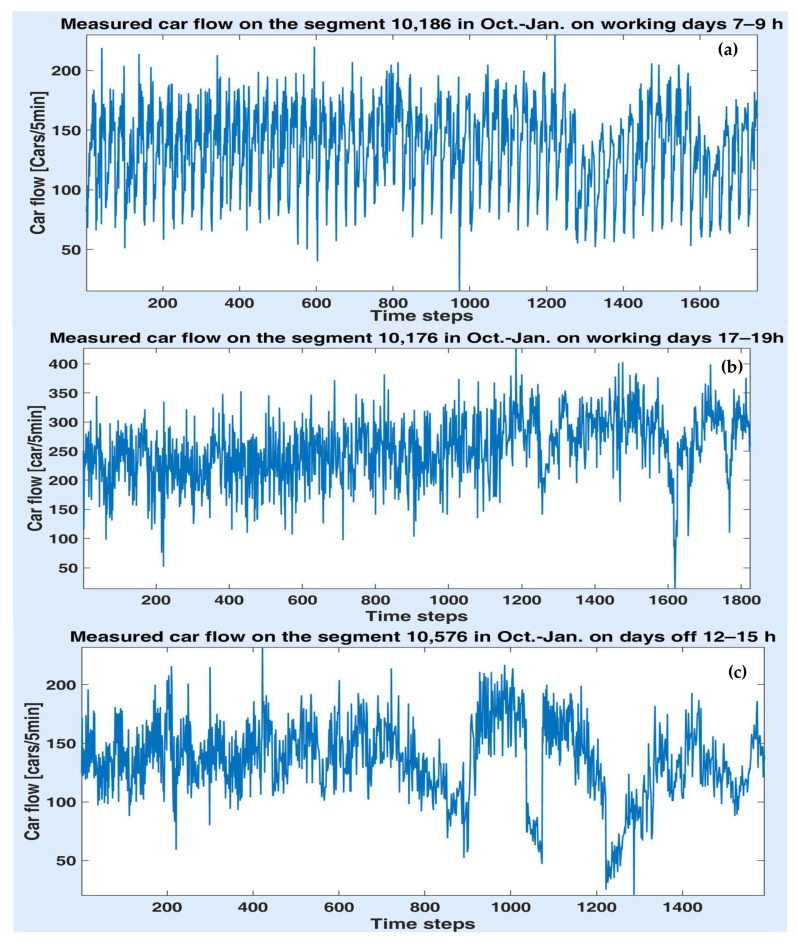

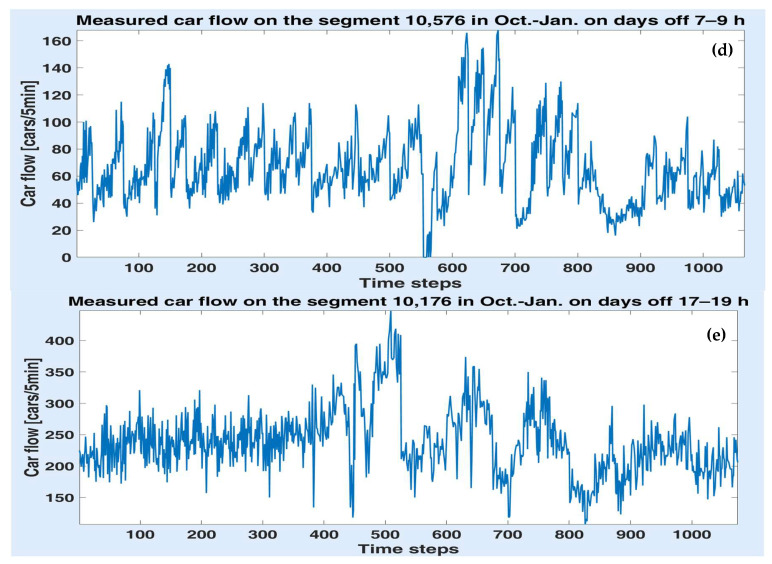
Measured car flow on the three segments on different days and time slots (**a**–**e**).

Figure 4 shows some visible differences between the traffic on the working days and the days off for all the segments. Consequently, in order to improve the prediction accuracy first, the data were separated into two categories: working days and days off. The days off include the weekends, children’s winter holidays and legal holidays (the National Day). Thus, the available datasets come from 73 working days and 44 days off. Furthermore, three time slots were considered for each day, regardless of the day type: 7–9 h, 12–15 h and 17–19 h, since the morning and evening (more precisely, afternoon) periods represent the rush hours in our city. For example, Figure 6a–e illustrates the variations of the car flow on different segments and time slots: segment 10,186 on the working days in the interval 7–9 h (a), segment 10,176 on the working days in the interval 17–19 h (b) and the segment 10,576 on the days off in the time slot 12–15 h. While in the time slot 7–9 h, the data seems to reveal a periodic variation, on the other two intervals, they are quite noisy.

Since the car flow was recorded over a period of 4 months, the number of available data should be large enough (tens of thousands, as presented in Table 1) to obtain accurate models.

The algorithms were implemented and run within the Matlab programming environment. The simulations obtained with the parametric model revealed that for a major part of the models, the best polynomial trend degree was null. The seasonal variation was identified especially for the working days and the time slot 7–9 h, and the structural indices of the ARMA model (na and nc) have varied in the range 10–80. The most accurate model was found from 20/30 generated configurations for the ARMA models by using the random search strategy. The algorithm was tested on datasets of 1500–2500 samples (corresponding to 65–70 working days and 43 days off, respectively) and performed in approximately 7/15 min. The prediction performance of each estimated model is shown in Table 2, Table 3 and Table 4 in terms of RMSE and MAE, corresponding to each route segment. One must mention here that the predicted values were computed by an iterative manner relying only on previously predicted values and measured data (as in (6)) and without taking into account the values observed between the prediction steps. In fact, one cannot have immediate access to the actual observations of the traffic data.

When applying the second approach, we used the same datasets as for parametric models, with 1500–2500 samples. The datasets were split into two subsets: the training and the test subset, respectively. The last 5 or 10 samples from the whole measured dataset represent the test subset and are left for evaluating the prediction accuracy (for the 5-step and 10-step prediction, respectively). The rest of the data represents the training subset. For each experiment, 30 training configurations have randomly been generated, depending on the number of the hidden units and the values of the hyper-parameters (the maximum number of epochs, the minimum batch size, the initial learning rate and the learning rate drop period). The best model configuration was selected by minimizing the variance of the model error computed with the help of the training subset. Thus, with the training dataset, we trained the network 30 times by randomly choosing the hyperparameter values (from a given set of values). This yielded 30 models. For each model, we computed the RMSE on the training horizon by using the difference between the simulated data and the training data. Then the model with minimum RMSE was chosen. With this optimal model, the next predicted values were computed. The RMSE on the prediction horizon is evaluated by using the prediction error, which means the difference between the predicted values and the test values. The algorithm was performed in approximately 12 min. The prediction performance metrics obtained with the best training model are shown in Table 2, Table 3 and Table 4 for each route segment. Some experiments were done on shorter datasets (e.g., the last 23 days off or the half of working days), but the prediction accuracy was lower.

Table 2 reveals a comparative analysis between the performances of the two methods in terms of RMSE and MAE on segment 10,176. Although most experiments denote that the LSTM model outperforms the ARMA model in both metrics, especially in the long term (50 min), sometimes the differences between the two approaches are quite small for short term prediction (e.g., for days off 12–15 h for 25 min, for 17–19 h for 50 min, working days 12–15 h for 50 min). Moreover, for some experiments, the ARMA model performs better than the LSTM model. (e.g., working days 7–9 h for 50 min, 12–15 h for 25 min).

In Table 3, the same comparative analysis in terms of RMSE and MAE is presented for segment 10,186. Furthermore, in this case, LSTM models are superior in prediction accuracy to the ARMA models in many cases, but for the working days, 12–15 h for 50 min, the models are quite close, and for the days off, 7–9 h for 25 min and 50 min the ARMA model performs better than LSTM model.

Table 4 presents the prediction performances of the models for segment 10,576. As usual, the LSTM models provide the smaller RMSEs and MEs than ARMA models. Still, the ARMA model performs better for the days off 7–9 h both for 25 and 50 min and 12–15 h for 25 and 50 min. Although one can note that there are small differences between the metric values of the models for working days 17–19 h for 25 min and working days 12–15 for 50 min, one cannot surely say that the models are equally performant. Some graphical inspections are necessary to reach a conclusion, as explained in the next section.

## 4. Discussion

Figures below depict the predicted values provided by the two models compared to the observed (actual) car flow values for each route segment, both on short and long time prediction horizon. Accordingly, the prediction errors are comparatively illustrated in the lower side of the figures, together with the prediction accuracy metrics (as in Table 2, Table 3 and Table 4). The superiority of a method to another is proved for different experiments both by the statistical metrics and the simulation results (the prediction error on the prediction horizon).

**Figure 7:**
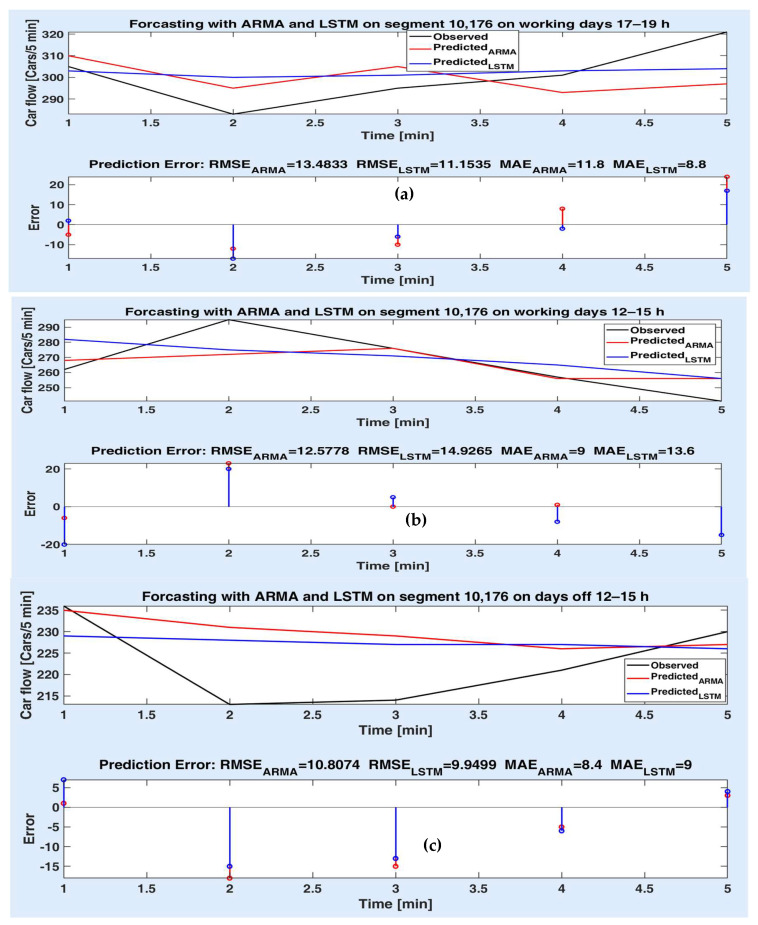
Measured vs. predicted car flows on the segment 10,176, on different days and time slot for 5–step prediction (25 min) (**a**) working days 17–19 h; (**b**) working days 12–15 h; (**c**) days off 12–15 h; prediction errors of both models.

Figure 7a–c illustrate the prediction performances obtained for the route segment 10,176, for 25 min. Figure 7a exemplifies the superiority of the LSTM model to the parametric model on working days and the time slot of 17–19 h. One can notice that the most values of prediction error are smaller for the LSTM model than for ARMA, including the last one (after 5 steps). This result is also highlighted by the smaller values of RMSEs and MEs. Although for most experiments, the LSTM model is superior to the ARMA model (as seen from Table 2) in prediction accuracy, Figure 7b denotes that the ARMA model can perform better (even slightly) than LSTM (e.g., for the time slot 12–15 h on working days), since the prediction error values are mostly smaller for ARMA model than for LSTM model, although after 5 steps, the prediction error values seem to be equal. There are few situations when both models perform approximately with the same accuracy. For example, in Figure 7c, one can note that the prediction errors of both models are very close to each other for each prediction step. Therefore, there are small differences between their corresponding metrics on the whole, 5-step prediction horizon.

**Figure 8:**
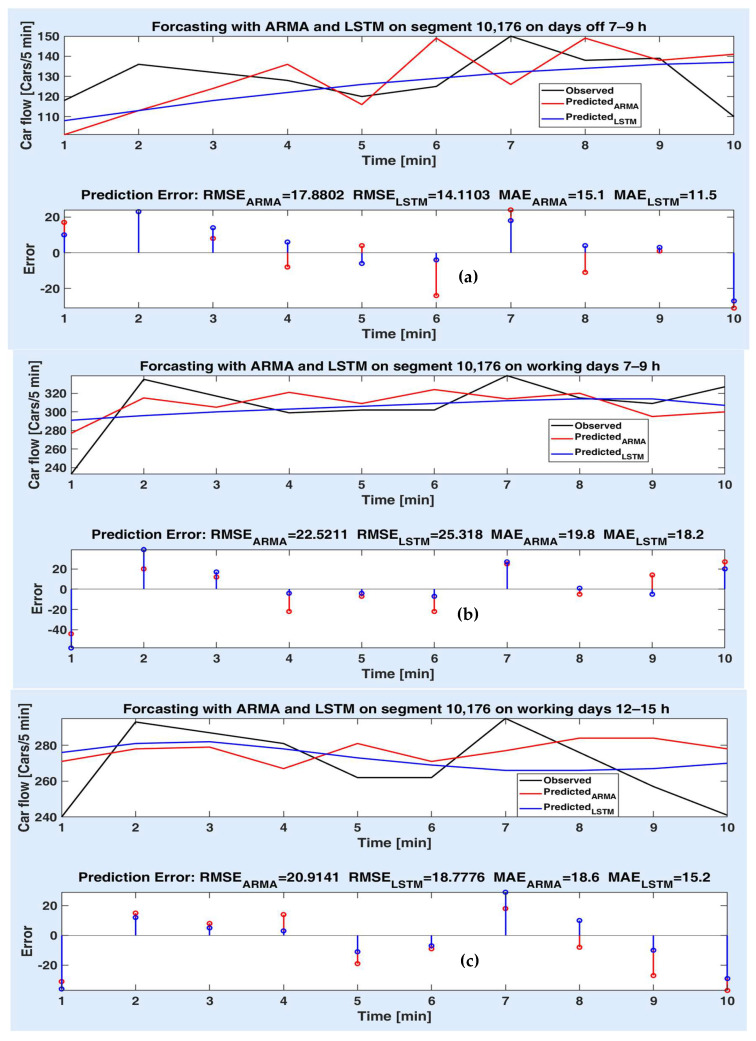
Measured and predicted values on segment 10,176 for 10 steps for (**a**) off days 7–9 h; (**b**) working days 7–9 h; (**c**) working days 12–15 h; prediction errors of both models different experiments.

Figure 8a–c depicts the prediction performances of the models on the route segment 10,176 for 50 min (10 steps ahead). In this case, the prediction accuracy is lower than for short-term prediction both for ARMA and LSTM model, as expected, due to the stochastic nature of the datasets. Moreover, recall that the predicted values are estimated based on the previously predicted values and not on the actual observations. A comparative analysis of the model prediction performances can also be realized in this case. Figure 8a shows that the LSTM model provides higher performance than the ARMA model on the whole prediction horizon. One can notice that the prediction error values are smaller than in the case of the ARMA model, and consequently, the RMSEs and MEs are smaller. Figure 8b reveals that the ARMA model can perform better than the LSTM model, although with a little higher differences from the observed data, especially at the first step. Figure 8c illustrates that the two models provide approximately the same prediction metric values, but at the first and the last step, they are quite far from the observed ones.

**Figure 9:**
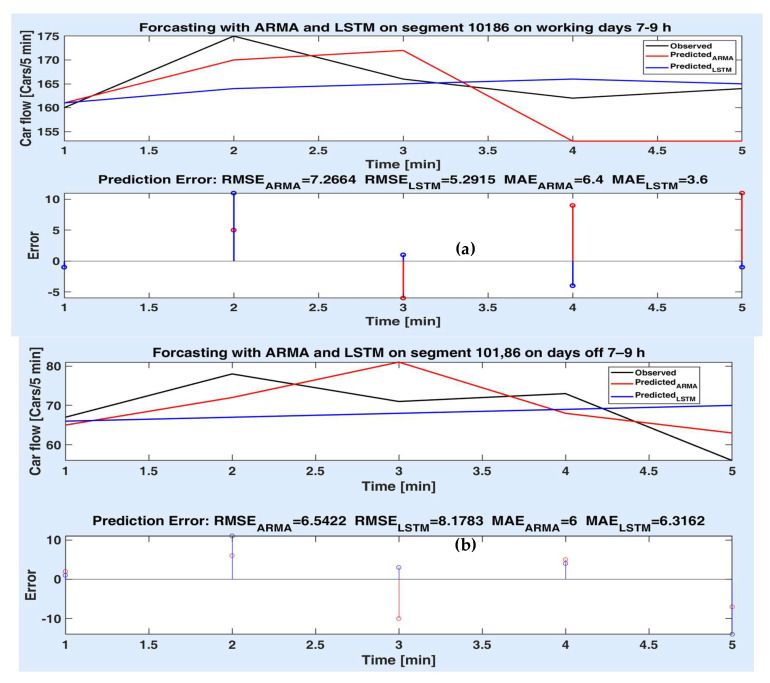
Measured and predicted values on segment 10,186 for 5 steps for (**a**) working days 7–9 h; (**b**) days off 7–9 h; prediction errors of both models.

Figure 9a,b illustrate the prediction performances obtained for the route segment 10,186, for 25 min. Figure 9a illustrates the advantage of the LSTM model to the parametric model since overall, the prediction error values of the LSTM model have smaller values, especially for the first and last sample. Figure 9b, on the contrary, indicates that, overall, the ARMA model is superior to the LSTM model, although the third value of the prediction error is bigger than for the LSTM model.

**Figure 10:**
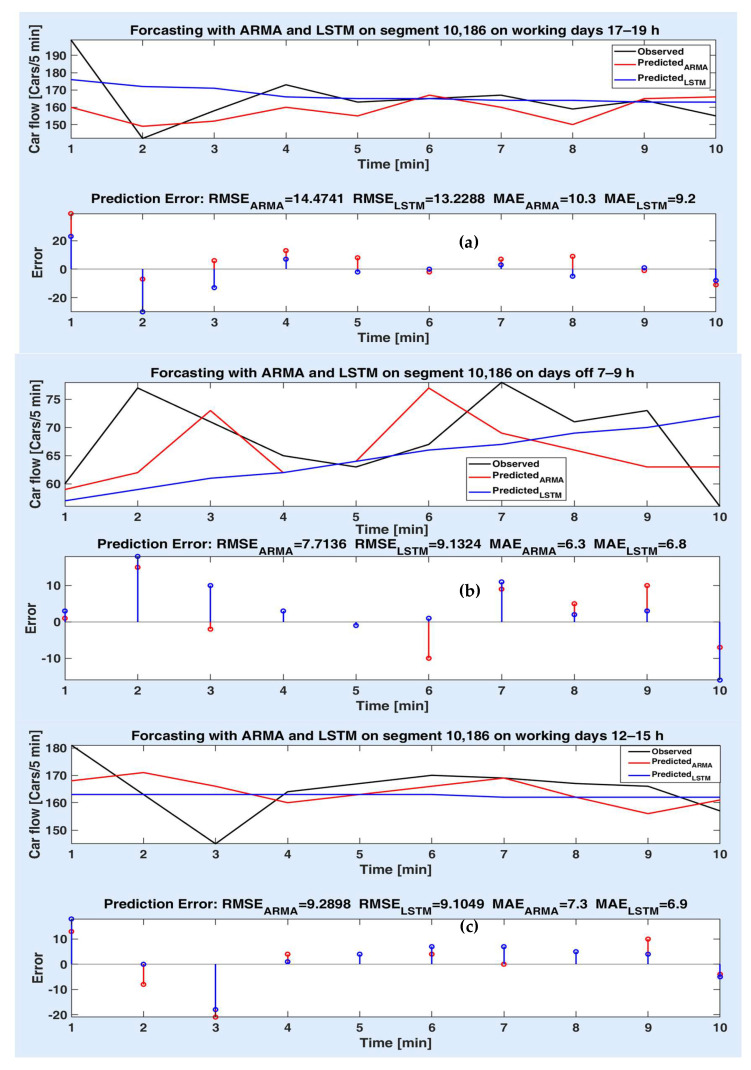
Measured and predicted values on segment 10,186 for 10 steps for (**a**) working days 17–19 h; (**b**) days off 7–9 h; (**c**) working days 12–15 h; prediction errors of both models different experiments.

Figure 10a–c illustrate the prediction performances obtained for the route segment 10,186 for a prediction horizon of 50 min. In Figure 10a, one can see that the prediction error values of the LSTM model are smaller than that of the parametric model over the whole prediction horizon, which leads to smaller metric values. Thus, one concludes that, in this case, the LSTM model performs better. Figure 10b instead shows that, overall, the ARMA model is superior to the LSTM model in prediction accuracy. Figure 10c represents a case when both models offer approximately the same result. One can note here that there are not many differences not for the prediction errors or for their performance metrics (9.28 vs. 9.1 and 7.3 vs. 6.9).

**Figure 11:**
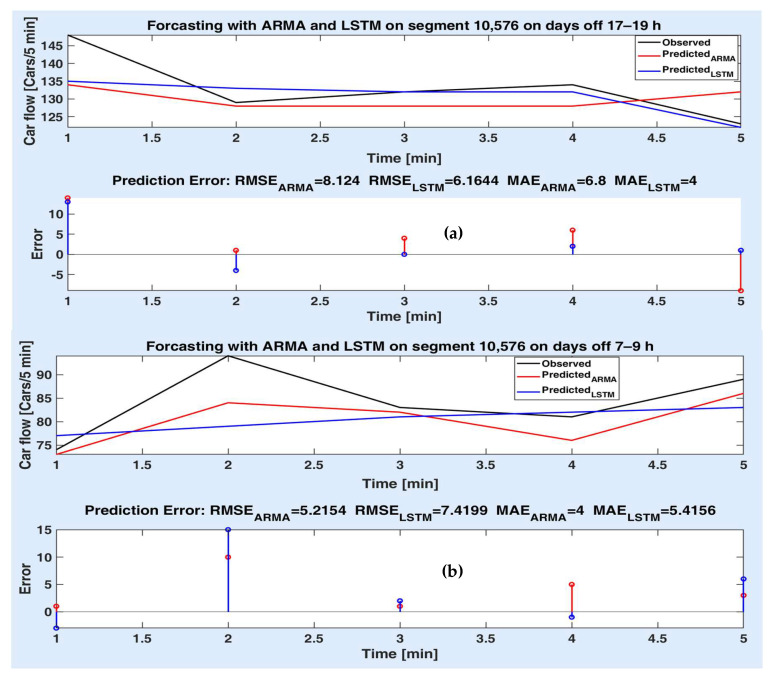
Measured and predicted values on segment 10,576 for 5 steps for (**a**) days off 17–19 h; (**b**) days off 7–9 h; prediction errors of both models’ different experiments.

Figure 11a,b illustrate the prediction performances obtained for the route segment 10,576, for a prediction horizon of 25 min. In Figure 11a, one can note that the LSTM model performs better than ARMA since the predicted values for the first model are mostly smaller than for the second one, including the first value (even quite far from the observed value). Figure 11b, on the contrary, proves the superiority of the ARMA model to the LSTM model in prediction performances, even with high accuracy.

**Figure 12:**
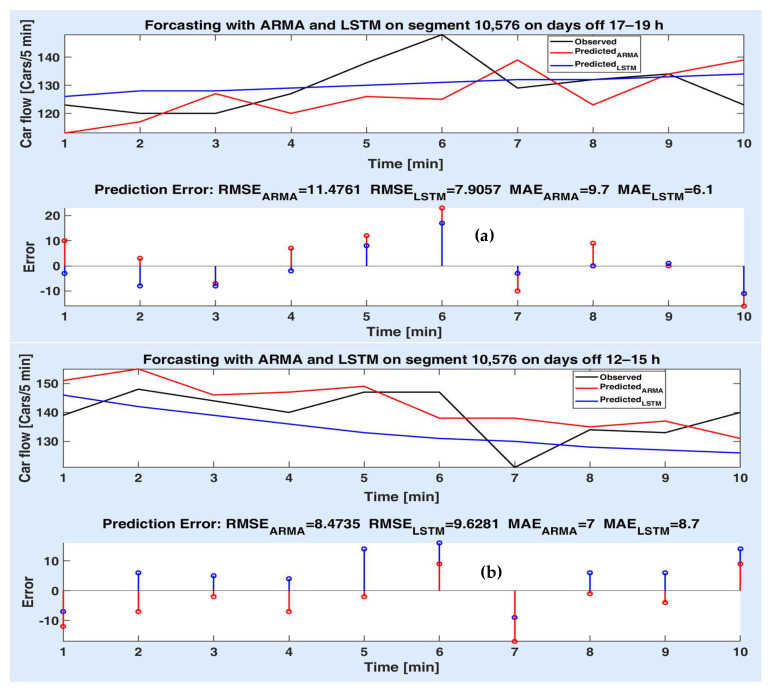
Measured and predicted values on segment 10,576 for 10 steps for (**a**) days off 17–19 h; (**b**) days off 12–15 h; prediction errors of both models’ different experiments.

Finally, Figure 12a,b depicts the simulation results for the route segment 10,576, for a prediction horizon of 50 min. In Figure 12a, one can note that the LSTM model performs much better than ARMA, including for the first and last value of the interval. Figure 12b can demonstrate that the ARMA model can lead to better prediction performances than the LSTM model since mostly the corresponding prediction errors are smaller for the ARMA than for the LSTM model.

One can conclude that the prediction accuracy depends not only on the statistical metrics (in this case, RMSE and MAE) but on the prediction error values. Usually, smaller values of the metrics represent higher prediction quality. Still, it can be suitable to complete the accuracy evaluation metrics with a visual analysis of the prediction error.

## 5. Conclusions

A neuro-inspired architecture for complex systems was presented, based on three control levels (the reflex action, learned behavior and strategic problem-solving). It was used for integrating, as a proof of concept, the urban traffic control subsystem of a smart city, thus testing the possibility to apply it on other smart city subsystems as a systemic approach for controlling such a complex structure.

The addressed urban traffic management system was centered on the avoidance of traffic congestion and designed as an agent-oriented model-based three-layered architecture. The real-time control layer, centered on junction traffic lights duration, is reactive to the car flow, as provided by sensors. In order to ensure not only the reactivity but also the proactivity of the traffic control, by means of perception function, the car flow prediction problem was investigated based on a real case study—the city of Bucharest—by employing two types of models: parametric (ARMA) and nonparametric (LSTM). Given the concept of the neuro-inspired architecture, it is important to determine if, at the level of learned behavior, the actual learning should center on parametric or nonparametric approaches.

The traffic flow prediction addresses a short (5 steps ahead) and a long (10 steps ahead) prediction horizon, which is covering the timing necessities of the architecture. The adjustment of traffic light models needs at least three steps in order to limit traffic congestion.

The two algorithms were tested on different size datasets, handling data acquired offline, but in order to increase the accuracy of prediction, the dataset needs to also be increased. Therefore, it is recommended to employ larger datasets, especially when they exhibit a strong variation (dynamic), although they correspond to the same time slot and day model. In both cases, the most adequate prediction models were identified by following a random strategy approach since it is known that it is the most efficient for real-time constraints (the runtime does not exceed 10–15 min). Therefore, the experiments included a fixed number of models, which allows for the possibility of not including the optimal one.

The simulation results and the statistical metric values showed that in the short term, both models could provide good prediction quality, but in the long term, the AI-based model mostly proved superior prediction accuracy to the ARMA model. Moreover, although we are mainly interested in predicting the car flow on the short-term horizon, the predicted values in the long term can be reliable, as well, and can be employed in the real traffic scenarios.

The predictive approach will be applied so that the changes made in the traffic light duration at the junction level are transmitted to the superior network control level while the entire system remains stable. In addition, this approach will help to determine the optimal number of junctions (i.e., the network structure) supervised by a model.

Further research in neuro-inspired control architecture can open a large set of research goals, as the possibility to adjust scenarios and agent behaviors by reinforcement learning, the integration of city events and of online traffic information from smart cars in the perception function for urban traffic, the possibility to define the functionality of human-centered smart transportation for smart cities, eventually correlated with smart building approach, integration of e-health in the smart city model, stepwise development of the smart global city open model a.s.o.

## Figures and Tables

**Table 1:** Statistics of the datasets.

Statistic	10,176	10,186	10,576	10,176 Work	10,176 Off	10,186 Work	10,186 Off	10,576 Work	10,576 Off
Count	33,696	33,696	33,696	21,024	12,672	21,024	12,672	21,024	12,672
Min	6	3	0	6	11	3	5	0	0
Max	520	300	359	520	48	300	23	359	29
Mean	190.34	106.64	117.77	203.86	167.71	114.28	93.91	128.38	100.15
St. Dev.	92.60	52.00	60.45	99.71	74.23	55.91	41.85	65.65	45.68
Variance	8574	2704	3654	9942	5510	3125	1751	4309	2086

**Table 2:** Prediction accuracy metrics for the segment 10,176.

Slot Time	Type	Model	RMSE (25′)	MAE(25′)	RMSE (50′)	MAE(50′)
7–9	Work	ARMA	15.74	14.4	22.52	19.8
7–9	Work	LSTM	13.22	12.4	25.31	18.2
7–9	Off	ARMA	17.63	13.4	17.88	15.1
7–9	Off	LSTM	14.19	10.4	14.11	11.5
12–15	Work	ARMA	12.57	9	20.91	18.6
12–15	Work	LSTM	14.92	13.6	18.77	15.2
12–15	Off	ARMA	10.8	8.4	25.37	21.5
12–15	Off	LSTM	9.94	9	17.91	12.1
17–19	Work	ARMA	13.48	11.8	17.94	14.4
17–19	Work	LSTM	11.15	8.8	12.71	9.01
17–19	Off	ARMA	15.77	12	24.17	21.7
17–19	Off	LSTM	12.41	10.32	23.96	20.7

**Table 3:** Prediction accuracy metrics for the segment 10,186.

Slot Time	Type	Model	RMSE (25′)	MAE(25′)	RMSE (50′)	MAE(50′)
7–9	Work	ARMA	7.26	6.4	14.64	10.8
7–9	Work	LSTM	5.29	3.6	15.51	10.8
7–9	Off	ARMA	6.54	6	7.71	6.3
7–9	Off	LSTM	8.17	6.31	9.13	6.8
12–15	Work	ARMA	8.08	7	9.28	7.3
12–15	Work	LSTM	4.22	3.4	9.1	6.9
12–15	Off	ARMA	9.57	6.4	10.29	7.9
12–15	Off	LSTM	6.88	6.71	8.87	5.6
17–19	Work	ARMA	6.48	4.4	14.47	10.3
17–19	Work	LSTM	5.78	4.22	13.22	9.2
17–19	Off	ARMA	10.62	10	16.53	15.1
17–19	Off	LSTM	8.34	6.3	14.31	10.05

**Table 4:** Prediction accuracy metrics for the segment 10,576.

Slot Time	Type	Model	RMSE (25′)	MAE(25′)	RMSE (50′)	MAE(50′)
7–9	Work	ARMA	21	17	21.58	17.3
7–9	Work	LSTM	16.35	11.6	18.77	13.8
7–9	Off	ARMA	5.21	4	12.07	8.9
7–9	Off	LSTM	7.41	5.41	14.73	10.4
12–15	Work	ARMA	9.04	7.8	13.05	11.6
12–15	Work	LSTM	7.62	6.6	11.85	10.4
12–15	Off	ARMA	9.12	6.4	8.47	7
12–15	Off	LSTM	10.98	8.6	9.62	8.7
17–19	Work	ARMA	12.81	10.2	20.33	16.8.
17–19	Work	LSTM	11.44	9.4	12.52	8.8
17–19	Off	ARMA	8.12	6.8	11.47	9.7
17–19	Off	LSTM	6.16	4	7.9	6.1
